# From Protection to Prevention: Redefining Vaccines in the Context of Antimicrobial Resistance

**DOI:** 10.7759/cureus.60551

**Published:** 2024-05-18

**Authors:** Mohammed Sallam, Johan Snygg, Doaa Allam, Rana Kassem

**Affiliations:** 1 Department of Pharmacy, Mediclinic Parkview Hospital, Mediclinic Middle East, Dubai, ARE; 2 Department of Management, School of Business, International American University, Los Angeles, USA; 3 Department of Management, Mediclinic City Hospital, Mediclinic Middle East, Dubai, ARE; 4 Department of Anesthesia and Intensive Care, University of Gothenburg, Sahlgrenska Academy, Gothenburg, SWE; 5 Department of Clinical Pharmacy, Queen's University, Belfast, IRL; 6 Department of Management, School of Business, University of Essex‎, Colchester, GBR

**Keywords:** vaccine development, overuse of antibiotics, infection‎, immunization, vaccine hesitancy, antimicrobial stewardship program, antimicrobial resistance‎, vaccines‎

## Abstract

Antimicrobial resistance (AMR) poses a significant threat to global health, compromising the effectiveness of treatments and increasing medical risks. In this crisis, the importance of vaccines in reducing AMR is being increasingly acknowledged, although not thoroughly explored. This literature review asserts that vaccines can significantly lessen the occurrence of infections, thereby reducing the need for antibiotics and limiting the emergence of resistance. Vaccines play a crucial role in antimicrobial stewardship programs by preventing diseases that would otherwise necessitate the use of antibiotics. Expanding vaccine coverage supports responsible usage of antimicrobials and aligns with global health priorities to maintain effective medical interventions. This review emphasizes the need for equitable funding and policy support for vaccine initiatives comparable to new antibiotics and diagnostic techniques. Moreover, it calls for more detailed investigations into vaccines' economic and health benefits in managing AMR, highlighting their potential as cost-effective solutions to this urgent health challenge. Through a careful analysis of existing literature, this review highlights the fundamental role of vaccines in transforming the landscape of AMR, shifting the focus from a protective approach to a preventive health strategy.

## Introduction and background

Antibiotics, within 120 years, have remarkably revolutionized contemporary medical practices and increased the typical human life expectancy by an additional 23 years [1]. Fleming's unexpected and successful discovery of penicillin in 1929 was quickly followed by the realization that improper drug use resulted in rapid bacterial resistance in both medical settings and the general population [2]. This resistance is a natural outcome of using antibiotics against microorganisms that have evolved to withstand threats to their survival.

Antimicrobial resistance (AMR) is a significant global health problem that poses substantial risks to modern medicine and public health [3]. A rise in resistance has the potential to render typical interventions of contemporary medicine, including surgery, chemotherapy, and cesarean sections, excessively unsafe to execute [4].

As bacteria, viruses, parasites, and fungi resist antimicrobial therapies, the number of effective antibiotics and other antimicrobial agents at our disposal decreases. Consequently, we become susceptible to infections that were previously easily treatable. Resistance to antimicrobials occurs naturally over time, usually through genetic changes. However, the misuse and overuse of antibiotics are speeding up this process.

In this context, there has been an increasing tendency in the last decade toward vaccines as a primary protection against AMR [5]. Therefore, the importance of immunizations is progressively acknowledged as a crucial approach to mitigating the escalation and dissemination of resistant microorganisms and is widely recognized as a complement to conserving existing antimicrobials [6]. The preventive characteristics of vaccines will not be subjected to the identical selection force endured by antibiotics. Utilizing vaccines can effectively manage and prevent the infection from occurring initially without encountering the challenges and dilemmas associated with treating the infection later on and the necessity for antibiotics to eliminate the disease [7]. Consequently, this proactive approach will contribute to safeguarding the whole population and specific vulnerable groups, such as the elderly or individuals with diabetes.

This review aims to address vaccines' capacity to significantly contribute to the battle against AMR. It advocates for a transition from merely managing AMR to actively preventing infections.

**Figure 1:**
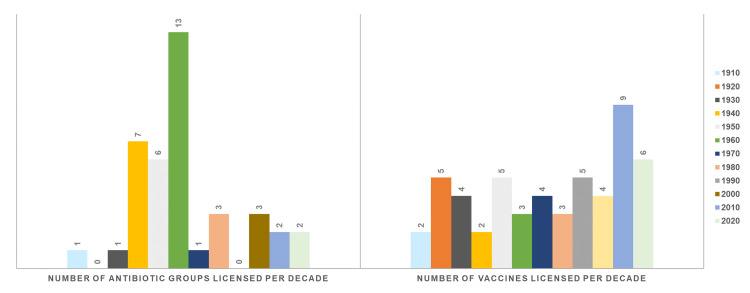
Historical trends in the licensing of antibiotic groups and vaccines by decade Information obtained from [1,8,9]‎ Graph Credit: Dr. Mohammed Sallam

Figure 1 illustrates the chronological evolution in the approval of antibiotic groups and vaccines throughout various decades, highlighting substantial patterns in medical progress. The licensing of antibiotics reached its pinnacle during the 1960s, commonly acknowledged as the "Golden Age" of antibiotic discovery, followed by a noticeable decrease [8]. Conversely, the creation of vaccines showcases a more diverse course, experiencing a significant surge in novel introductions during the 2010s due to intensified global health efforts and advancements in vaccine research technology [9]. This visual representation emphasizes the fluctuating nature of medical breakthroughs and the evolving emphasis on public health priorities over the preceding century.

## Review

Understanding AMR

AMR is one of the critical health challenges of the 21st century. Globally, the threats of resistance are increasing alarmingly [10].‎ According to recent estimates, the average annual deaths due to AMR will exceed those due to cancer by 2050 [10]. Additionally, it is predicted that the economic consequences resulting from AMR could reach a staggering USD 100 trillion, potentially causing a decline of up to 4% in the global annual gross domestic product in an extreme situation [11].

Microorganisms, including bacteria, viruses, fungi, and parasites, are some of the planet's most versatile and successful life forms. They have been around for over 3.5 billion years and have survived in the harshest conditions [12]. Today's microorganisms are no different, and their resilience and adaptability have allowed them to become one of the greatest threats to the health of humans and animals. This widespread AMR problem can occur in all forms of invasive pathogenic microbes. The Centers for Disease Control and Prevention (CDC) has listed many pathogens concerning drug resistance patterns [13]. The abuse and mismanagement of antibiotics in humans are among the main causes that have led to AMR [2].

Considering the rapid evolution of resistance toward every newly introduced class of antibiotics and the difficulties in creating effective new drugs, it is not enough to concentrate solely on research on the fundamental resistance mechanisms and the development of new antibiotics. Hence, adopting an integrated approach incorporating innovative interventions is crucial to effectively tackling AMR [14].

**Figure 2:**
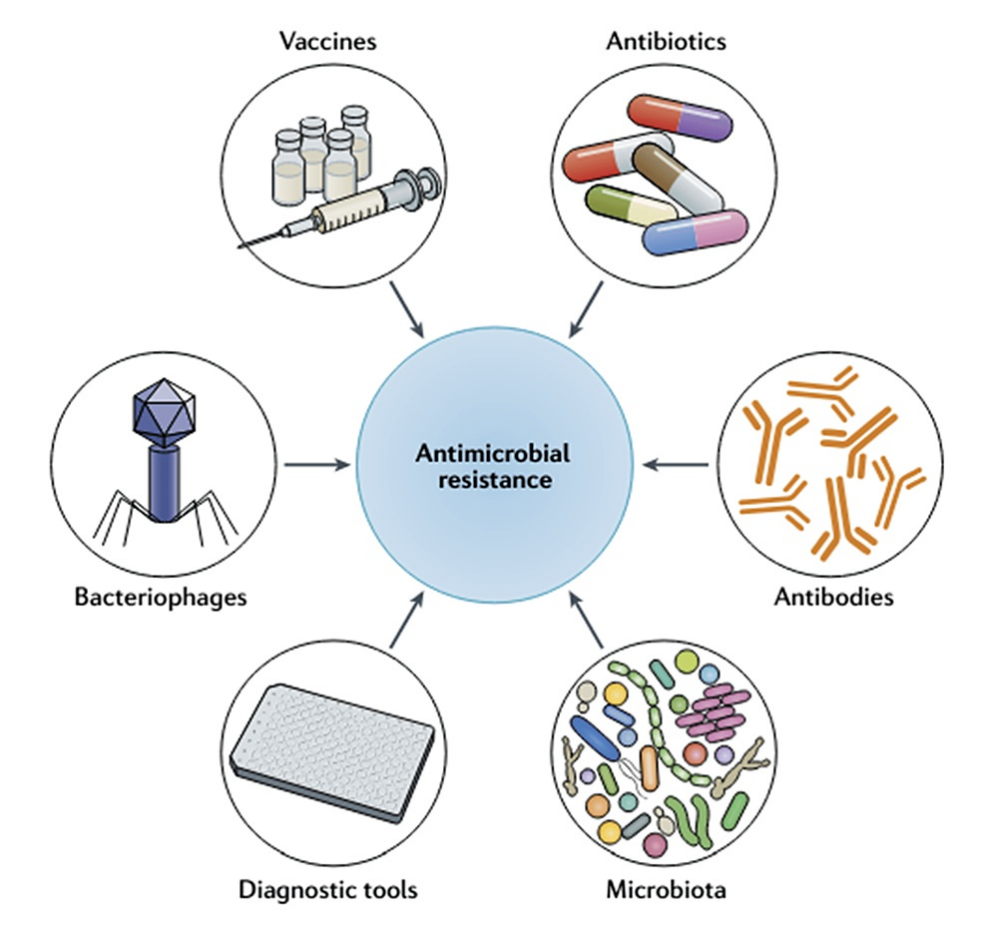
Innovative approaches to combat AMR AMR: antimicrobial resistance Image Source: Micoli et al., 2021 [15]

Figure 2 showcases a range of techniques and instruments utilized in the battle against AMR, which is a significant and escalating menace to global health. The diagram outlines six crucial elements: vaccines, antibiotics, bacteriophages, antibodies, diagnostic tools, and microbiota. Each component has a distinct purpose: averting infections, directly combating harmful pathogens, or supplying vital data to ensure successful treatment.

Importance of vaccinations in AMR management

In the past, vaccines and antimicrobial agents have been considered separately in discussions about combating infectious diseases. However, when their potential impact on controlling disease is compared, it can be argued that a case should be made for AMR to be considered when making decisions about the use of vaccines [16]. This argument is because the more that infection can be prevented by vaccination, the less likely antibiotics will be used. The importance of vaccination has recently emerged with the COVID-19 pandemic, for which the fast development of safe and efficient vaccination was an urgent necessity.

Vaccines have been fantastic public health tools for reducing the disease burden. Few people would disagree with including vaccines on lists of the most important public health measures of the 20th century.

Optimizing antimicrobial stewardship programs to confine antimicrobial use and related expenses is urgent [17]. Therefore, the administration of vaccines has been emphasized as an essential element in the effort to decrease AMR and is a crucial aspect of a comprehensive antimicrobial stewardship strategy [18]. Vaccines can directly and indirectly reduce the emergence and transmission of AMR. Initially, a vaccine targeting a specific bacterial pathogen decreases the prevalence of antibiotic-resistant pathogens and consequently reduces the use of antibiotics [19]. The pneumococcal vaccine provides the most extensively documented example of this effect [20]. Numerous studies indicate that a decrease in the carriage and infections caused by the pathogen among vaccinated individuals significantly reduces antibiotic prescriptions and hinders the circulation of resistant strains [7].

**Figure 3:**
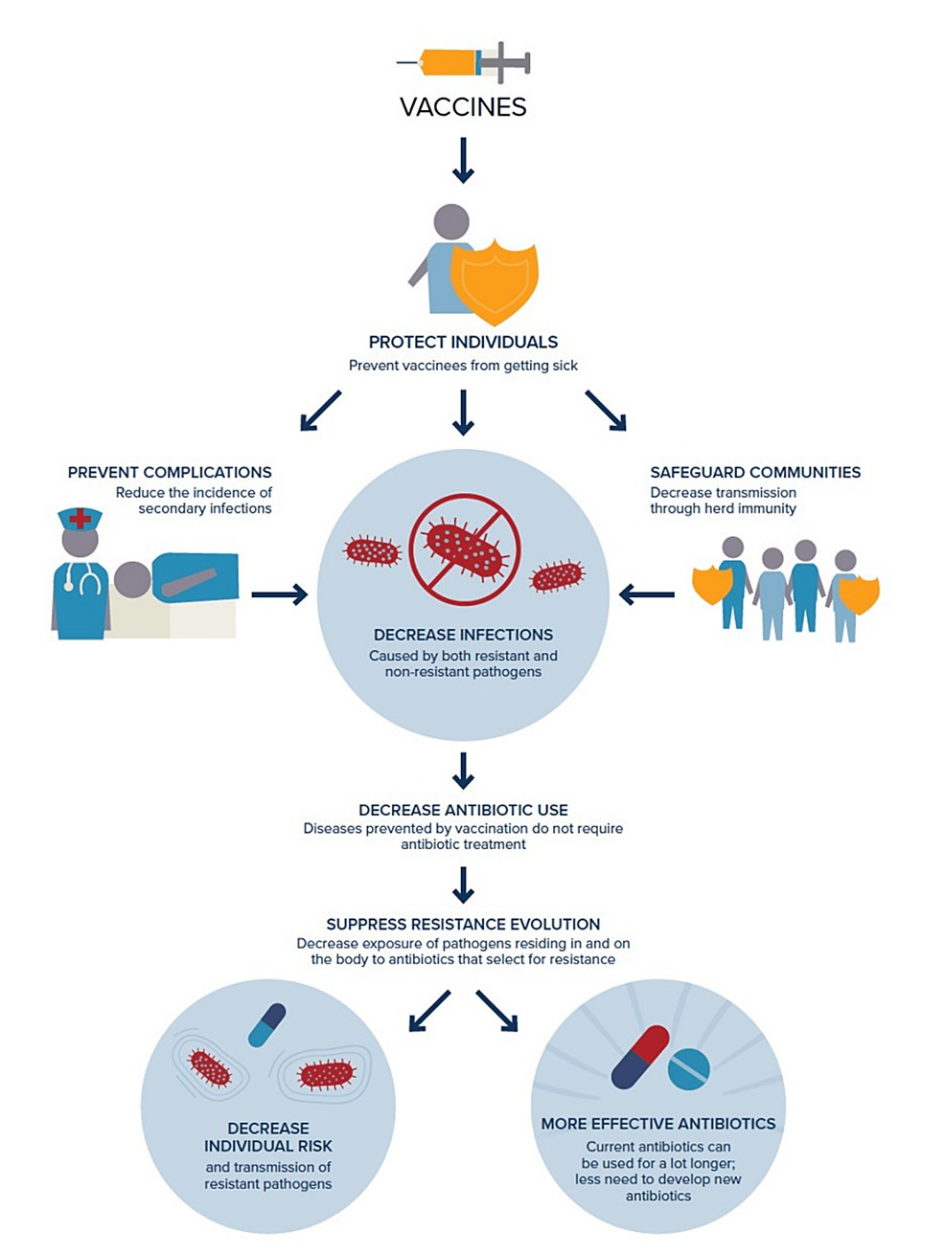
An illustrative pathway showing the influence of vaccines on AMR AMR: antimicrobial resistance Image Source: The Annex to Immunization Agenda 2030 by the World Health Organization 2020 [19]

Figure 3 presents a flowchart illustrating the diverse influence of vaccines in addressing AMR. The chart delineates a progressive system of advantages wherein vaccines are pivotal in averting infections.

Vaccines and other immunotherapies can be powerful tools to reduce the burden of AMR by preventing infections [21]. Any infection that can be prevented through vaccination is a scenario in which the overall burden of antibiotic-resistant diseases is reduced. The likelihood of unfavorable outcomes is mitigated by eradicating the need for antibiotic treatment. Avoiding antibiotics minimizes the chances of promoting the development of resistant strains of the intended pathogen and other susceptible species [22].

Table 1 shows specific pathogens where vaccines and other immunotherapies could help reduce the AMR burden.

**Table 1:** Overview of selected antimicrobial-resistant pathogens with their vaccine and AMR ‎status‎ AMR: antimicrobial resistance, CDC: Centers for Disease Control and Prevention Source: Adapted from [2,12,13]

Pathogen	AMR Status	CDC priority	Vaccine Status	Vaccine Action on AMR
Streptococcus pneumoniae	Common resistance to several antibiotics	Serious	Conjugate vaccines widely used, broader protection vaccines under investigation	Direct and bystander effects
Methicillin-resistant *Staphylococcus aureus* (MRSA)	Resistance was observed and reported for β-Lactams, aminoglycosides, tetracyclines, macrolides, glycopeptides, quinolones, lipopeptides, and oxazolidinone	Serious	No licensed vaccines	Bystander effects expected
Pseudomonas aeruginosa	Resistance was observed and reported for β-Lactams, aminoglycosides, quinolones, and polymyxins	Serious	No licensed vaccines	Bystander effects expected
*Haemophilus influenzae* type b (Hib)	Widespread prior to vaccine introduction	Not specified	Conjugate vaccine licensed and widely used	Direct and bystander effects
Vibrio cholera	Growing single and multidrug resistance	Not specified	Oral vaccine licensed and widely used	Mostly direct
Salmonella typhi	Recent global expansion of resistant lineages. Resistance observed and reported for β-lactams, sulfonamides, chloramphenicol and fluoroquinolones	Serious	Several vaccines are available, with conjugate vaccines showing promise	Direct and bystander effects
Mycobacterium tuberculosis	Growing single and multidrug resistance. Resistance was observed and reported for β-Lactams, fluoroquinolones, aminoglycosides, macrolides, lincosamides, p-aminosalicylic acid, and pyrazinamide.	Serious	Live BCG vaccine with variable efficacy, new vaccines under development	Direct
Neisseria meningitidis	Some resistance to antibiotics	Urgent	Vaccines against most main serogroups (A, B, C, W, Y)	Direct
Clostridium difficile	Infection associated with antibiotic use. Resistance was observed and reported for Aminoglycosides, β-lactams, tetracyclines, macrolides, glycopeptides, and quinolones.	Urgent	Vaccines in development, monoclonal antibodies licensed	Direct, bystander, and selective targeting effects
*Escherichia coli* (UPEC & ETEC), uropathogenic *Escherichia coli* (UPEC), or enterotoxigenic *Escherichia coli* (ETEC)	AMR is a significant concern for UPEC treatment. Resistance observed and reported for β-Lactams (including carbapenems), aminoglycosides, tetracyclines and quinolones	Urgent	Vaccine candidates in development	Direct and bystander effects
Group A *Streptococcus*	Resistance was observed and reported for tetracycline and macrolides	Concerning	No licensed vaccines	Bystander effects expected
Klebsiella pneumoniae	Multidrug resistance is endemic in many areas. Resistance was observed and reported for β-Lactams (including carbapenems), aminoglycosides, and fluoroquinolones	Urgent	No vaccines tested in humans	Bystander effects expected
Neisseria gonorrhoeae	Increasing AMR is a serious concern in some cases. Resistance was observed and reported for tetracyclines, β-lactams (including extended-spectrum cephalosporins), fluoroquinolones, sulfonamides, and spectinomycin	Urgent	No vaccine, potential protection from N. meningitidis type b vaccine	Bystander effects expected
Influenza virus	Some resistance to antivirals	Not specified	Several licensed vaccines are widely used	Bystander and interspecific effects
Rotavirus	No antivirals in use	Not specified	Several licensed vaccines are widely used	Bystander effects
Norovirus	No antivirals in use	Not specified	Vaccines in clinical and preclinical trials	Bystander effects
Respiratory syncytial virus (RSV)	Not a concern	Not specified	Vaccines under clinical trial. Monoclonal antibody products are available to protect infants and young children from severe RSV.	Bystander and interspecific effects
Dengue virus	No antivirals in use	Not specified	Partially protective licensed vaccine	Bystander effects
*Plasmodium falciparum* (malaria parasite)	Some resistance to antimalarials	Not specified	Partially protective licensed vaccine	Direct

Vaccines as a tool for reducing antibiotic use

Vaccines can predispose to infections that require antibiotics. An example of this is when children have ear infections. Prior to the heptavalent pneumococcal conjugate vaccine (PCV7) licensure, it was estimated that otitis media was the most common reason for pediatric antibiotic prescriptions. With the significance of pneumococcal serotypes in mind, it is estimated that in children under 24 months, the vaccine effectiveness in preventing otitis media or acute otitis media ranged from 8% to 42.7%. In children under 60 months, the vaccine effectiveness ranged between 13.2% and 39%, which eradicates the need for antibiotics regarding these infections [23].

Childhood vaccination against *Streptococcus pneumoniae* and rotavirus has also been linked to a lower likelihood of requiring antibiotic treatment [7]. Vaccinations targeting infectious agents like *Streptococcus pneumoniae* and *Haemophilus influenzae* have previously demonstrated a decreased tolerance toward antimicrobial medications [24]. The implementation of universal free influenza immunization in Ontario, Canada, resulted in a substantial 64% reduction in the number of prescriptions for respiratory antibiotics associated with influenza [25].

These findings underscore the potential of vaccination as a critical strategy for reducing antibiotic utilization and as a positive contribution to the fight against antibiotic resistance.

Enhancing immune response through vaccinations

The most significant means by which vaccines counteract AMR is through the immunity they provide [26]. Vaccines allow the immune system to identify antigens particular to the pathogens they are designed for, and sometimes even to specific pathogen variations. On the other hand, antibiotics target standard bacterial functions found in numerous species of microorganisms, both harmful and harmless. As a result, vaccines generally do not impact the overall evolution of microorganisms, except for the specific strains they target. In contrast, antibiotics can exert selective evolutionary pressure on both targeted and non-targeted microorganisms, leading to the development of resistance. Therefore, it is highly likely that resistance will emerge over time, even with the creation of new generations of antibiotics [2]. The specificity of vaccines allows for potential strategies that cannot be achieved with antimicrobials, let alone nonpharmaceutical approaches. For instance, vaccines can target highly pathogenic strains of a particular pathogen specifically. Although antibiotics can cure diseases faster than vaccines, they do not change disease exposure and are unlikely to reduce severe complications. On the other hand, immunization with vaccines prevents diseases and can reduce the need for antibiotics because there may not be a need for treatment by preventing the diseases.

It is traditional for people to consider how vaccines are directly effective against diseases they prevent. While this is important, surveillance studies have shown that herd immunity from vaccines may provide longer-lasting protection against specific diseases than efforts using antimicrobial treatments [27]. Now that vaccines can be specially designed to target the specific antigens of a microbe, vaccines can offer far superior immunity to antimicrobial drugs and antibiotics, given that the latter are becoming increasingly less effective against prevalent serious pathogens [26].

This preventative aspect of immunization, which reduces the prevalence of diseases that would otherwise be treated with antibiotics, is highly significant in reducing selective pressure for AMR.

Challenges and limitations in utilizing vaccinations

Frequent challenges and limitations are currently impeding the recognition of vaccines and the successive decrease in antimicrobial utilization. These factors negatively impact vaccination's success and global effects on AMR. They include vaccine development and distribution, vaccine hesitancy and misinformation, and access to and affordability of vaccines.

Vaccine Development and Distribution

Vaccine development and distribution is an ever-changing field based on global health needs and advances in new technology [9]. There are few licensed bacterial vaccines and none specifically for AMR pathogens. This gap is partly due to a perceived lack of financial incentives for vaccine manufacturers. Previous vaccines designed to combat common infections, such as the pneumococcal conjugate vaccine, have substantially reduced the incidence of resistant disease [20]. The potential economic and health benefits of preventing and controlling AMR by targeting specific pathogens or high-risk populations are starting to be recognized, and it is hoped that as resistance increases in severity, more vaccines will be developed to target AMR infections specifically. However, many currently available vaccines have been developed mainly for use in Western countries with comparatively low resistance rates. More must be done to ensure that these vaccines effectively prevent resistant infections in other parts of the world where the disease burden is higher.

The emergence of new pathogens and antibiotic-resistant bacteria drives the need for new vaccine technologies. Given these changes, established methods for identifying new vaccine candidates are insufficient to ensure global protection. Hence, new vaccine technologies that can achieve rapid development and large-scale production are of pivotal importance [28].

Vaccine Hesitancy and Misinformation

Considering the potential emergence of pandemics, vaccine hesitancy is quickly becoming a recognized threat to international health [29]. While vaccines have helped notably reduce sickness and death from a variety of infectious diseases, there are now individuals who refuse or allow their children to be vaccinated.

Vaccine hesitancy and misinformation involve delays in the acceptance or refusal of vaccines, despite the availability of vaccination services. Healthcare workers have ranked this as the most common reason people do not have vaccines on many continents. This uncertainty may be based on many factors, including risk perception, religious or philosophical beliefs, and vaccine misinformation. Many studies have explored the acceptance of the measles, mumps, and rubella vaccine. A study from Jordan found a concerning level of hesitancy and resistance, with trust in vaccine safety and efficacy, behavior, and having fewer offspring being associated with acceptance [30]. Another study from the Kingdom of Saudi Arabia revealed that COVID-19, seasonal influenza, and monkeypox vaccination conspiracies correlated with higher reported side effects and negative attitudes toward booster and mandatory vaccinations [31].

Access and Affordability of Vaccines

It is estimated that about 20.5 million children worldwide are still not accessing life-saving vaccines [32]. Several issues deter the accessibility and affordability of vaccines and have considerable implications for depleting antibiotic use with the use of vaccines [33]. The high cost of vaccines is of substantial importance to the global accessibility of vaccines. However, discrepancies in determining the actual price of a vaccine and the cost-effectiveness of vaccines compared to the treatment of diseases can influence decision-making patterns for vaccine use [34]. A study among Jordanian healthcare workers evaluated influenza vaccine uptake and showed that two-thirds were willing to receive the vaccine if provided for free [35]. Countries that offer free vaccines for their population have protocol guidelines in use by their governmental health policymakers.

Vaccine Payers' Exclusion

Various factors influence vaccine coverage and availability, including economic barriers, conflict situations, and payers' reluctance to include immunizations in the patient's benefits scheme. The decrease in vaccination rates and the rise in exemption rates render the general population more vulnerable to disease outbreaks that vaccines can prevent [36].

Vaccine coverage is required to ensure population protection. Removing the cost barrier will likely increase vaccination rates, especially among adolescents and adults, and for newer, more expensive vaccines. Also, it will incrementally increase children's immunization rates. It will also introduce opportunities for public health, medical practitioners, health plans, and employers to develop creative approaches to immunizing more people once the cost barrier is removed.

So, additional efforts by all stakeholders to reach important subpopulations (e.g., by age group) will be needed. This strategy would best involve all essential stakeholders and private health plans: employers who purchase health insurance for their employees, providers who deliver health services, public health leadership, vaccine manufacturers, and patients with their families; they share common goals but very different approaches and constraints [37].

Improving vaccine access and enhancing vaccine research and development

Developing vaccines for diseases primarily affecting low-income countries and ensuring equitable access is crucial for global health and equity. Research should focus on these diseases and vaccines suitable for these populations. Innovative approaches like a global trust fund for health technologies could incentivize research in these areas [38]. A coordinating facility can align vaccine research priorities and prevent duplication. Pricing structures should be affordable for those in extreme poverty while preventing leakage to more prosperous groups. Competition among vaccine producers can lower prices and increase availability. Strengthening health systems and capacity building are essential for countries with weaker immunization programs. Utilizing vaccines fully can have a significant impact on global health and address the threat of AMR. Integrated efforts and lessons from successful interventions can improve vaccine access for the poorest populations. Investing in vaccine access will have a double dividend for global health.

A significant but often neglected area of vaccine research is a better understanding of the immunology of vaccine-preventable diseases and the immune responses to vaccines [26]. This information is critical for the development of new vaccines, for improving the efficacy of current vaccines, and for the appropriate use of vaccines in different populations. This type of research training must be supported by scientists in both the developed and developing worlds.

Research should be maintained to develop new and improved vaccines and determine the optimal usage and delivery of available vaccines [28]. Evaluation of vaccines with new adjuvants, delivery systems, or schedules should be supported if there is an indication that these will lead to significant improvements in efficacy, safety, or the cost of immunization. Cost-effectiveness is always an important consideration and should be examined in the context of both the direct and indirect costs of immunization and the cost of not vaccinating [39]. These studies should consider the needs and acceptability of potential end users of new and improved vaccines in the developed and developing worlds.

Ongoing and advanced research is necessary to develop more effective, safer, and less costly vaccines [39,40]. Research should be accelerated for diseases where vaccines do not yet exist and for vaccines that require multiple doses and have low coverage rates. Development should also be guided by the specific needs of low-resource countries with a high burden of infectious diseases.

Study limitations

There are some limitations regarding the studies used as the basis for the current literature review. The differences in how the studies were designed and their overall quality could impact the strength and applicability of the conclusions drawn. Also, it is essential to note that the evidence presented only covers a limited number of vaccines and does not encompass all available ones. Additionally, some studies may not reflect the most up-to-date research findings due to the continuously evolving nature of vaccine technology and AMR. Finally, the review may not have fully considered the impact of socioeconomic and cultural factors on vaccine acceptance and effectiveness.

Future strategies and recommendations

Evidence-based strategies to increase immunization are desperately needed. Economic pathways determine the value of reduced AMR. To accurately measure these pathways, it is best to utilize models that can assess the long-term macroeconomic benefits of vaccines in reducing AMR. Health economic evaluation, encompassing cost-effectiveness and cost-utility/benefit analyses, is commonly employed in several developed nations to evaluate the expenditures and advantages of vaccination programs. It may be advantageous to broaden the scope of this evaluation to encompass long-term indirect outcomes, such as the role of vaccination in reducing AMR [25].

Additionally, research into barriers to immunization and strategies to reach under-immunized subpopulations within these age groups will continue vital work on disparities in immunization rates [2]. Identifying new ways to engage providers, payers, and employers in increasing immunization rates may also be promising [37].

It is essential to continually stress the importance of research into new and improved vaccines against a broader range of diseases and encourage the pharmaceutical industry to produce these vaccines, considering that vaccines in a free market economy are less profitable than drugs [16].

Lastly, artificial intelligence has emerged as a potent tool in healthcare. Therefore, AI-powered decision support systems can aid in optimizing antimicrobial stewardship programs by delivering real-time guidance on appropriate antimicrobial selection, dosing, and duration based on patient-specific factors and local resistance patterns [41].

## Conclusions

The advantages of immunization and vaccines are extensively acknowledged, emphasizing the necessity of implementing methodologies that encourage their utilization in combating AMR. Crucial to these efforts is guaranteeing cost-efficiency and broadening the availability of vaccines. In the current economic environment, every healthcare strategy, including interventions related to vaccines, must exhibit economic sustainability. Given that AMR persists as an escalating threat, our strategies should prioritize prevention by taking full advantage of the proactive ability of vaccines to decrease the occurrence of communicable diseases.

To effectively incorporate vaccines into antimicrobial stewardship programs, it is essential to enhance public and professional understanding. This process includes building trust in healthcare providers, removing financial barriers, addressing safety concerns, and improving communication strategies. Such actions are crucial for developing public health policies and reducing the burden of AMR, thus preserving the efficacy of existing treatments and ensuring a healthier future.
